# Supplementary material for: Patterns of Socioeconomic Inequities in SDGs Relating to Children’s Well-Being in Thailand and Policy Implications
Source: Int J Environ Res Public Health. 2022 Oct 20;19(20):13626. doi: 10.3390/ijerph192013626 (PMC9603103; doi:10.3390/ijerph192013626)
Supplement: Supplementary file 1 [file ijerph-19-13626-s001.zip › ijerph-1877616-supplementary.pdf]

Supplementary Materials

Table S1. Matched the child flourish index indicators with available of the Thai MICS 2019 data and outcomes

The child flourish index				The MICS 2019	
**Domain**	**SDG indicator**		**Thailand's score***	**Data availability**	**Outcome (unweighted)**
Under 5 survivals	SDG 3.2.1	Under-five mortality rate (deaths per 1,000 live births)	1.0	Not available	
Maternal survival	SDG 3.1.1	Maternal mortality ratio (maternal deaths per 100,000 live births)	1.0	Not available	
Access to maternal & child health services	SDG 3.b.1	Proportion of the target population covered by DTP3 (%)	1.0	Available: IM5, IM6DTP1D, IM6DTP1M, IM6DTP1Y, IM6DTP2D, IM6DTP2M, IM6DTP2Y, IM6DTP3D, IM6DTP3M, IM6DTP3Y	95.32%
	SDG 3.1.2	Proportion of births attended by skilled health personnel (%)	1.0	Available: MN19	99.76%
Safe & clean environments	SDG 6.2.1.a	Prevalence of unsafe or unimproved sanitation (%)	1.0	Available: WS11, WS15	2.34%
	SDG 3.6.1	Deaths due to road injuries for ages 0-19 years	0.25	Not available	
Lack of extreme poverty	SDG 1.1.1	Children living in households in extreme poverty (%)	1.0	Applied, used the 1st quintile income household. Available: WINDEX5	28.40%
	SDG 1.2.1	Children living below the national poverty line (%)	0.5		
Educational achievement	SDG 4.1.1	(Aligned with) Expected years of school by age 20-24 years	0.75	Not available	
	SDG 4.1.1	(Aligned with) Harmonized mean test scores for age 15-19 years	0.5	Not available	
Mental health	SDG 3.4.2	Suicide mortality rate for per 100,000 population for ages 15-24	1.0	Not available	
Growth & nutrition	SDG 2.2.1	Prevalence of stunting among children under 5 (%)	0.5	Available: HAZ2	12.70%
	SDG 2 & 3	Children born low birth weight per 100 live births	0.5	Available: AN12BU, AN12BN	9.58%
Female agency & opportunity	SDG 3.7.2	Adolescent birth rate (births per 1,000 girls 10-19)	0.75	Available: WB4, CM11	136.60/1,000<br>13.7%
	SDG 5.3.1	Proportion of women aged 20-24 who were married or in union before age 15 (%)	0.75	Available: WB4, WAGEM	6.81%
Protection from violence	SDG 16.2.3	Proportion of young women & men aged 18-29 experienced sexual violence by age 18 (%)	0.5	Not available	
	SDG 5.2.1	Proportion of ever-partnered women & girls aged 15-49 subjected to physical, sexual or psychological violence by a current or former intimate partner in the past 12 months (%)	0.5	Not available	

*Score: 0.01 = very poor, 0.25 = poor, 0.5 = neither poor nor adequate, 0.75 = adequate, 1.0 = good [2,7]

Table S2. Independent and dependent variables details

	Question	Question number	Category	
	Wealth index quintiles	WINDEX5	poorest (1), poor (2), middle (3), rich (4), richest (5)	
	Area	HH6	municipal/urban (1), non-municipal/rural (2)	
	Region	HH7	Bangkok (1), central (2), north (3), northeast (4), south (5)	
	Language of household head	LANGUAGE	Thai language (1), and non-Thai language (2)	
	Religion of household head	HC1A	Buddhism (1), Islam (2), Christianity (3), others (6), no religion (7)	
	Highest level of education attended	WELEVEL (indivudual women), MELEVEL (maternal education), HELEVEL (household's leader education)	pre-primary or no school attended (0), primary school (1), lower secondary school (2), upper secondary school (3), higher education (4)	
	Sex	HL4 (individual person), HHSEX (household leader's sex)	male (1), female (2)	
			**Yes (1)**	**No (0)**
	Who assisted with the delivery of the last-born baby in the last 2 years?	MN19	doctor, nurse/midwife, or practical nurse/nurse's aid	other valid answers
	Vaccination document had seen	IM5	all of questions (date of vaccination) had to be completed with only the health handbook seen, or only other documents seen (IM5=1, 2)	incomplete date of vaccination or no document evidence
	Date of DTP vaccination	IM6DTP1D, IM6DTP1M, IM6DTP1Y, IM6DTP2D, IM6DTP2M, IM6DTP2Y, IM6DTP3D, IM6DTP3M, IM6DTP3Y		
	What kind of toilet facility do members of your household usually use?	WS11	used open pit or did not have facility/ used bush or field or sharing with others who are not members of the household	other valid answers without sharing with others who are not members of the household
	Do you share this facility with others who are not members of your household?	WS15		
	Wealth index quintiles	WINDEX5	lowest wealth index quintile (WINDEX5=1)	other valid answers
	Height for age by z-score (WHO median)	HAZ2	height-for-age <-2SD of the WHO Child growth standards median (HAZ2= -5.99 to -2.01)	other valid answers
	What was the birth weight of the child (kilograms)?	AN12B	birth weight< 2.500 grams with health handbook evidence (AN12BU=1)	other valid answers with health handbook evidence
	Birth weight evidence (health handbook)	AN12BU		
	Number of children who were born with each woman	CM11	had at least 1 child (CM11>=1)	no child (CM11=0)
	Age at first marriage/union of woman	WAGEM	marriage before 15 (WAGEM<15)	other valid answers

Figure S1. Result of unweighted proportion of the poorest households (national weighted), and GINI coefficient by regions

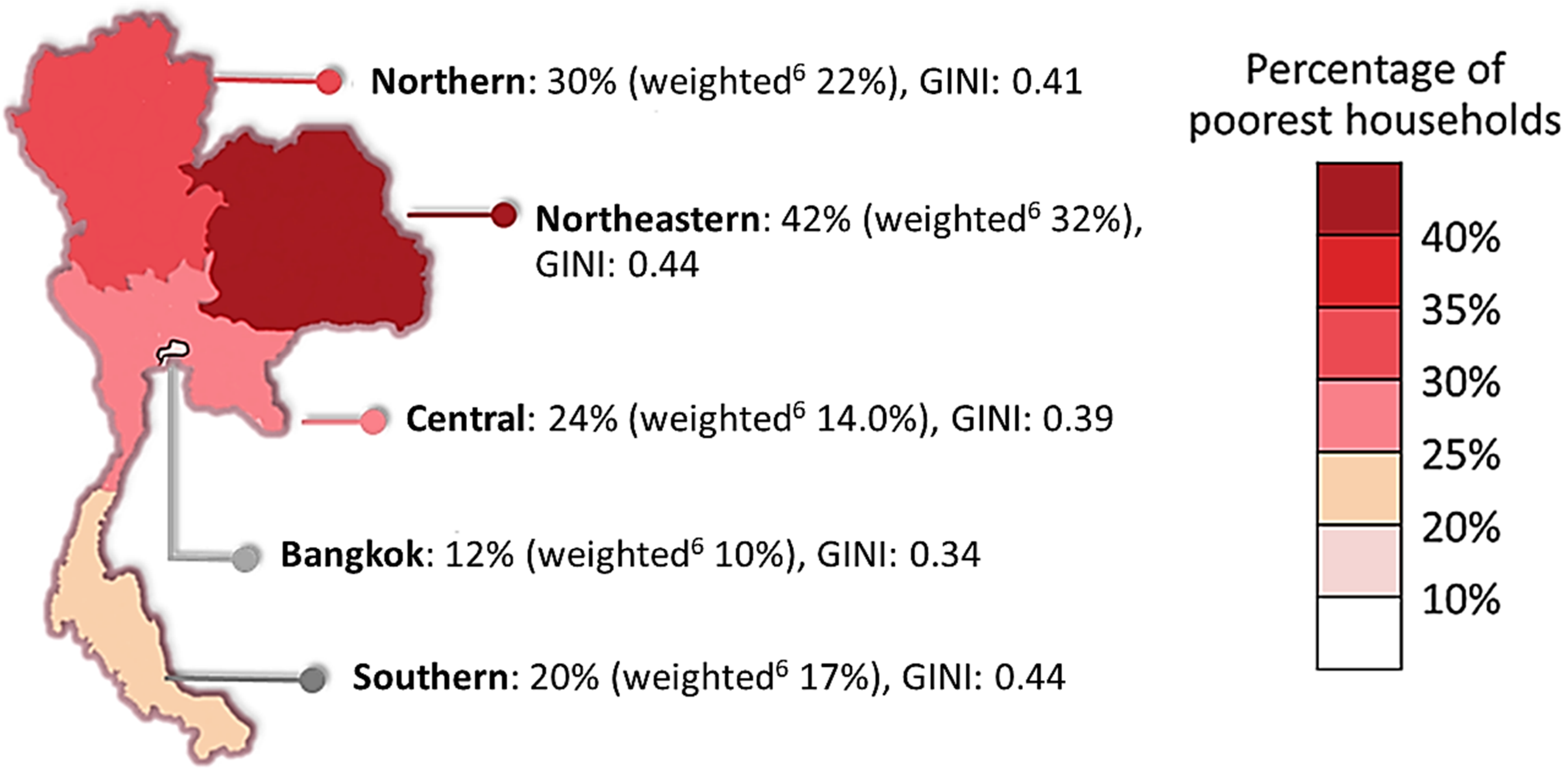

GINI coefficient illustrated income disparities, range between 0-1. Higher score means higher income disparities.

Data from National Statistical Office, Thailand. Income distribution. Gini coefficient of Consumption Income by Region and Area: 1988 - 2019 Available online: http://statbbi.nso.go.th/staticreport/page/sector/en/08.aspx (accessed on 8 April 2022).
